# LEF1-AS1 Deregulation in the Peripheral Blood of Patients with Persistent Post-COVID Symptoms

**DOI:** 10.3390/ijms26104806

**Published:** 2025-05-17

**Authors:** Alisia Madè, Santiago Nicolas Piella, Marco Ranucci, Carlo Gaetano, Laura Valentina Renna, Rosanna Cardani, Gaia Spinetti, Valentina Milani, Fabio Martelli

**Affiliations:** 1 Molecular Cardiology Laboratory, IRCCS Policlinico San Donato, Via Morandi 30, 20097 San Donato Milanese, Italy; alisia.made@grupposandonato.it (A.M.); sn.piella@uninsubria.it (S.N.P.); 2 Department of Cardiovascular Anesthesia and ICU, IRCCS Policlinico San Donato, Via Morandi 30, 20097 San Donato Milanese, Italy; 3 Laboratorio di Epigenetica, Istituti Clinici Scientifici (ICS) Maugeri IRCCS, 27100 Pavia, Italy; carlo.gaetano@icsmaugeri.it; 4 BioCor Biobank, IRCCS-Policlinico San Donato, Via Morandi 30, 20097 San Donato Milanese, Italy; lauravalentina.renna@grupposandonato.it (L.V.R.); rosanna.cardani@grupposandonato.it (R.C.); 5 Laboratory of Cardiovascular Pathophysiology and Regenerative Medicine, IRCCS MultiMedica, 20138 Milan, Italy; gaia.spinetti@multimedica.it; 6 Laboratory of Biostatistics and Data Management, Scientific Directorate, IRCCS Policlinico San Donato, 20097 San Donato Milanese, Italy; valentina.milani@grupposandonato.it

**Keywords:** COVID-19, long COVID, noncoding RNAs, long noncoding RNAs, microRNAs, major physical symptoms, major neurological symptoms

## Abstract

Long COVID denotes the persistence of symptoms after acute SARS-CoV-2 infection lasting for at least two months without another identifiable cause. Affecting an estimated 15% of COVID-19 patients, long COVID manifests in a wide range of symptoms. Despite extensive research on its one-year effects, limited data exist beyond 12 months. Due to the different manifestations of long COVID, its diagnosis can be challenging. Identifying potential mechanistic contributors and biomarkers would be highly valuable. Recent studies have highlighted the potential of noncoding RNAs (ncRNAs) as biomarkers for disease stratification in COVID-19. Specifically, we have recently identified miR-144-3p and a subset of lncRNAs as candidates for assessing disease severity and outcomes in COVID-19. This nested case–control study extends such investigations to 98 long COVID patients recruited 18 months after hospitalization, exploring the relationship between circulating ncRNA expression and persistent symptoms. While miR-144-3p, HCG18, and lncCEACAM21 expression did not differ between symptomatic and asymptomatic patients, LEF1-AS1 was downregulated in the peripheral blood mononuclear cells (PBMCs) of symptomatic patients. Of note, multiple LEF1-AS1 isoforms and LEF1 sense transcript levels were reduced and negatively correlated with relevant clinical markers. While further studies are needed, our discoveries offer new perspectives on the diagnosis and management of persistent long COVID.

## 1. Introduction

Long COVID, also known as post-COVID, is a multisystem condition defined as the continuation or emergence of new symptoms three months after SARS-CoV-2 infection, persisting for at least two months without any other identifiable cause [1]. The World Health Organization (WHO) has estimated that 10–20% of patients who suffered from COVID-19 develop long COVID symptoms [2]. Although some studies suggest that COVID-19 vaccination reduces the risk of developing long COVID symptoms [3,4,5], it remains a significant global issue [6]. This challenge is partly due to unequal access to vaccines and booster doses. Despite some studies indicate that the severity of COVID-19 infection may have an impact on the probability of developing long COVID symptoms [7,8], a European Respiratory Society statement reported that the risk factors for long COVID are not necessarily associated with the initial severity of the illness. Instead, they could be linked to female sex, age, and the number of symptoms experienced at onset [9,10]. Common symptoms of long COVID include fatigue, shortness of breath, and cognitive impairment, but the condition is highly variable, affecting numerous organs [11,12]. The pathogenesis of long COVID is not yet fully understood, but it is thought to involve a combination of persistent viral infection, immune dysregulation, and residual tissue damage. These factors may contribute to the diverse range of symptoms experienced by patients [11].

Although numerous studies have investigated the long-term effects of COVID-19 one year after infection [13], mainly focusing on pulmonary functions and respiratory outcomes [14,15,16] and cognitive deficits [17], there are limited data on the condition beyond 12 months from the onset of COVID-19 [5,18,19,20,21].

Due to the different manifestations of long COVID, the diagnosis of the syndrome can be challenging. Hence, identifying potential mechanistic contributors and biomarkers is highly valuable. In line with this evidence, noncoding RNAs (ncRNAs) have shown great promise as biomarkers for various diseases, including COVID-19. ncRNAs can be classified as small noncoding RNAs (e.g., microRNA) or long noncoding RNAs (lncRNAs), based on their transcript size. ncRNAs can regulate the transcription and translation of protein-coding RNAs [22] and are also detectable in the peripheral blood, showing potential as disease biomarkers.

In previous studies investigating acute patients, we explored the expression levels of a specific microRNA (miRNA), miR-144-3p, in plasma or serum samples from three different groups of COVID-19 patients recruited across Europe, including both hospitalized and non-hospitalized individuals. qPCR analysis identified miR-144-3p as a promising candidate for risk-based stratification and mortality prediction in COVID-19 illness [23]. Among the lncRNAs, HCG18-family lncRNAs, lncCEACAM21, and LEF1-AS1-202 lncRNAs are downregulated in the PBMCs of non-surviving COVID-19 patients compared to surviving ones, as well as in critical compared to severe patients, thus emerging as potential candidates for discriminating severity and predicting mortality rates in hospitalized COVID-19 patients [24]. Accordingly, a machine-learning model based on age and lncRNA LEF1-AS1 can predict the outcomes of both hospitalized and non-hospitalized COVID-19 patients in large groups of European and Canadian patients [25].

Focusing on hospitalized patients, in this study, we investigated the expression of selected COVID-19 ncRNAs in a cohort of 98 long COVID patients enrolled 18 months from COVID-19 hospitalization and assessed their potential role as biomarkers for the presence of long COVID symptoms.

## 2. Results

### 2.1. Long COVID Patient Characteristics

For this study, 98 patients hospitalized at IRCCS Policlinico San Donato because of mild to severe COVID-19 were enrolled in the “long COVID” protocol to assess symptoms persistent after hospitalization. To be enrolled in this protocol, patients had to physically return to the hospital, which may have led to the exclusion of individuals with more severe long COVID symptoms. Table 1 summarizes the general characteristics of the patients during their acute phase, including COVID-19 pre-existing conditions and information about in-hospital treatments. While most patients required oxygen therapy during their stay in the hospital, only 2% of the entire population required admission to the intensive care unit (ICU). In agreement with a previous study performed at our hospital also analyzing very long COVID subjects, patients were classified for the presence or absence of major physical symptoms (MPSs) or major neuropsychological symptoms (MNSs) [5], as assessed at follow-up. MPSs were more common among older patients, former smokers, and those with a history of atrial fibrillation or hypertension. Conversely, they were less frequent in patients who did not require oxygen therapy during hospitalization, possibly indicating a milder form of acute illness (Table 1). Persistent MNSs were more frequently observed in older individuals and those who had pre-existing diabetes at the time of having acute COVID-19 (Table 1).

After a median of 18 months from the onset of COVID-19, patients were enrolled in the long COVID protocol, peripheral blood was harvested, and patients were asked about the presence or development after discharge of symptoms, recorded in Table 2.

Among symptoms still present at the time of follow-up, the most frequent were persistent fatigue (38%), dyspnoea (34%), and memory dysfunction (30%). 

### 2.2. No Difference in Plasma miR-144-3p Levels in MPS or MNS Long COVID Patients

To investigate whether the development or the absence of long COVID symptoms was associated with the persistent dysregulation of plasmatic miR-144-3p [23], its expression levels were measured in platelet-poor plasma samples from long COVID patients. Results revealed no significant differences in miR-144-3p expression levels in the comparison of patients with or without MPSs or MNSs (Figure S1).

### 2.3. Lower LEF1-AS1 Expression Levels in the PBMC of MPS Patients

**Figure 1:**
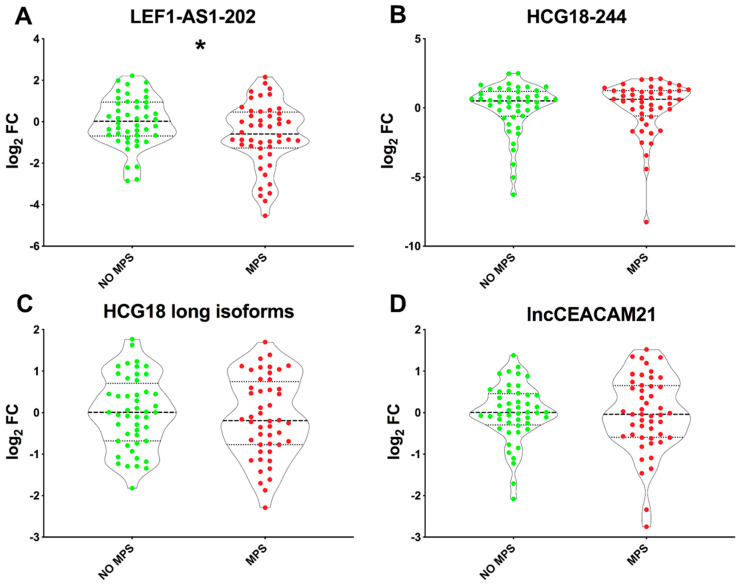
**Lower LEF1-AS1-202 levels in patients with major physical symptoms (MPSs) compared to no MPS patients.** The levels of LEF1-AS1-202 (**A**), HCG18-244 (**B**), HCG18 long isoforms (**C**), and lncCEACAM21 (**D**) were measured in RNA extracted from PBMCs derived from patients with or without MPS (MPS, *n* = 48; no MPS, *n*  =  46–48). LEF1-AS1-202 (**A**) showed a significant decrease in MPS patients compared to patients with no MPSs (* *p* ≤ 0.05), while no significant differences were observed in the expression levels of the HCG18-244 (**B**) or HCG18 long isoforms (**C**) or lncCEACAM21 (**D**). Values are represented as log_2_ fold changes and shown using violin plots, with dashed lines indicating the median and dotted lines denoting the second and third quartiles. An unpaired *t*-test (two groups) was used for statistical comparison.

To determine whether COVID-19 lncRNA biomarkers [24] remain persistently dysregulated, potentially indicating the development of long COVID symptoms, RNA extracted from PBMC samples from long COVID patients was analyzed. LEF1-AS1-202 displayed significantly decreased levels in MPS patients compared to those without MPSs (Figure 1A) but not in patients affected by persistent MNSs compared to their controls (Figure S2A). Conversely, the expression of HCG18 (both HCG18-244 and long isoforms) and lncCEACAM21 displayed no significant differences between patients with or without MPSs (Figure 1B–D), or MNSs (Figure S2B–D). Thus, LEF1-AS1 expression in the PBMCs of long COVID patients was further explored.

### 2.4. LEF1-AS1 Isoforms and LEF1 Are Similarly Deregulated in PBMCs of Long COVID Patients

**Figure 2:**
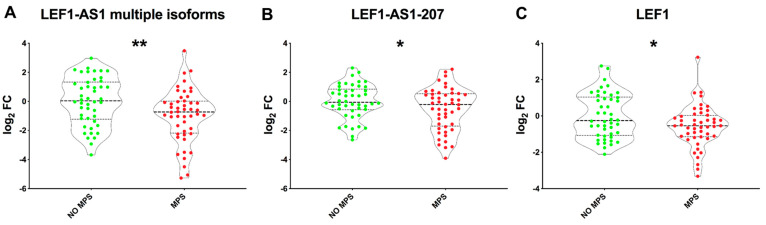
**Lower levels of LEF1-AS1 isoforms and LEF1 in patients with major physical symptoms (MPSs).** LEF1-AS1 multiple isoforms (**A**), LEF1-AS1-207 (**B**), and LEF1 (**C**) were measured in total RNA extracted from the PBMCs of patients with or without MPS (MPS, *n* = 48; no MPS, *n*  =  46). Values are represented as log_2_ fold changes and shown using violin plots, with dashed lines indicating the median and dotted lines denoting the second and third quartiles. An unpaired *t*-test (two groups) was used for statistical comparison. * *p* ≤ 0.05; ** *p* ≤ 0.01.

Different LEF1-AS1 isoforms are annotated in GRCh38.p14 (Figure S3). In line with LEF1-AS1-202 modulation, other LEF1-AS1 isoforms were investigated in long COVID patients. Specific primers were designed to detect the LEF1-AS1 -210, -211, -204, -212, and -209 isoforms collectively (referred to as LEF1-AS1 multiple isoforms) or the LEF1-AS1-207 isoform. Additionally, to validate possible co-regulation with LEF1, its expression levels were also analyzed. Decreased levels of all LEF1-AS1 isoforms tested were observed in patients affected by MPSs compared to their no-MPS controls (Figure 2A,B), consistent with the LEF1-AS1-202 result. Furthermore, LEF1 expression levels were also lower in patients with MPSs compared to those without MPSs (Figure 2C), indicating a similar regulation of LEF1-AS1 and LEF1.

**Figure 3:**
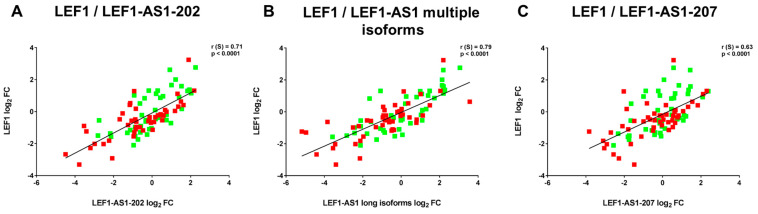
**LEF1-AS1-202, LEF1-AS1 multiple isoforms, and LEF1-AS1-207 positively correlate with LEF1 expression levels.** Correlations for LEF1-AS1-202 (**A**), LEF1-AS1 multiple isoforms (**B**), and LEF1-AS1-207 (**C**) were calculated using Spearman’s correlation test. Coefficients and *p* values are indicated. Red and green squares indicate patients with major physical symptoms (MPSs) (*n*= 48) and no MPSs (*n* = 49), respectively.

Accordingly, LEF1 levels were correlated positively with the expression of LEF1-AS1-202 (r(S) = 0.71, *p* < 0.0001), of LEF1-AS1 multiple isoforms (r(S) = 0.79, *p* < 0.0001) and of LEF1-AS1-207 (r(S) = 0.63, *p* < 0.0001) (Figure 3).

As for the LEF1-AS1-202 isoform, no significant differences were observed in the expression levels of all other LEF1-AS1 isoforms and of LEF1 when patients were grouped according to MNSs (Figure S4).

A univariate logistic regression model was used to examine the relationship between the dependent variable (MPSs or no MPSs) and the expression levels of all the LEF1-AS1 isoforms and LEF1. Figure S5 shows that a decrease in both the different isoforms of LEF1-AS1 and LEF1 was associated with a higher probability of MPSs.

### 2.5. LEF1-AS1 Isoforms and LEF1 Correlated with Relevant Clinical Parameters

Clinically relevant hematological tests were conducted in the peripheral blood harvested at follow-up from long COVID patients (Table S1).

Although still within reference values, patients with MPSs exhibited significantly higher white blood cell levels (*p* < 0.0007) and lower lymphocyte levels (*p* < 0.03). Conversely, no significant differences were observed in the comparison of clinical parameters between patients with or without MNSs.

**Figure 4:**
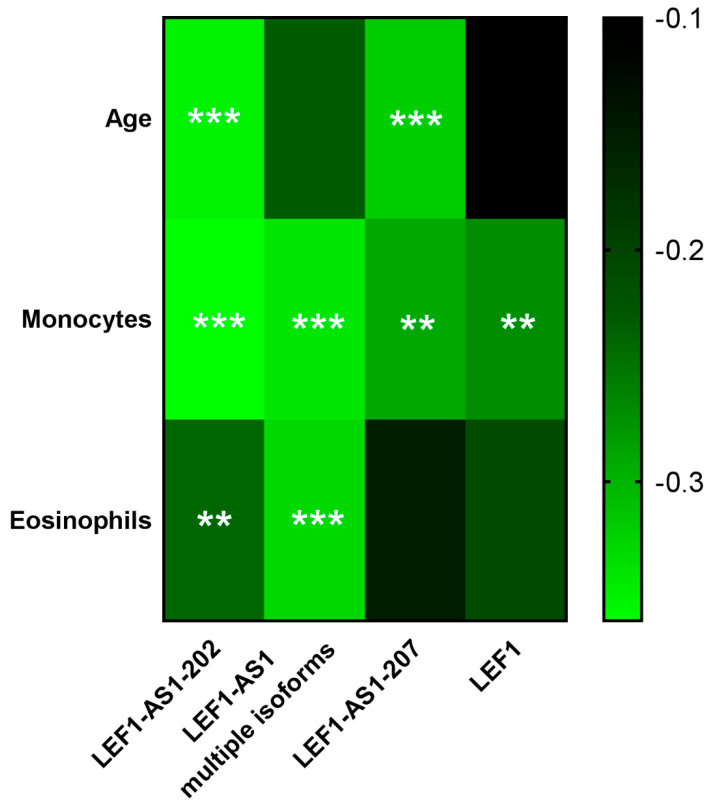
**Heat-map displaying the correlations of LEF1-AS1 isoforms and LEF1 with clinically relevant parameters.** Clinical parameters were measured at the moment of the interview; a brighter green color indicates a stronger negative correlation while a red color indicates a positive correlation. R coefficients were calculated with Spearman’s correlation test, and stars indicate statistical significance (** *p* ≤ 0.01; *** *p* ≤ 0.001; *n* = 97).

Interestingly, some weak but significant correlations were identified (Figure 4 and Supplementary Figure S6): LEF1-AS1 and LEF1 were negatively correlated with monocyte and eosinophil levels; moreover, LEF1-AS1-202 and -207 were inversely correlated with the age of long COVID patients. This prompted us to perform a multiple logistic regression analysis of LEF1-AS1 and LEF1 regulation, adjusting for age and being a former smoker, i.e., the main variables not directly related to COVID-19 that were significantly associated with the outcome in the univariate analysis (Table 1, *p* < 0.01). Following this adjustment, LEF1-AS1-202 and -207 did not display significant expression differences, in keeping with their inverse correlation with age, while the dysregulation of LEF1-AS1 multiple isoforms remained significant and LEF1 displayed borderline (*p* = 0.06) statistical significance (Table S2).

## 3. Discussion

Given the wide range of long COVID symptoms, identifying potential mechanistic elements and biomarkers for long COVID is necessary to enhance the clinical evaluation and management of affected patients. Some investigations suggest that the likelihood of experiencing long COVID symptoms decreases over time [26], but multiple studies report that the incidence of long COVID symptoms remains high even more than a year after the acute phase of COVID-19 [5,27,28,29]. These findings align with the symptom incidence observed in our patient cohort, in which the most common reported symptoms include cognitive issues, sensorimotor disturbances, shortness of breath, and fatigue, all of which significantly affect daily life. Specifically, patients were classified according to specific disease categories (MPSs and MNSs) to investigate specific aspects of long COVID.

In this study, we evaluated the expression of a subset of ncRNA biomarkers in long COVID patients, enrolled at a median of 18 months post-hospitalization for COVID-19. Specifically, we assessed the possible modulation of miRNA and lncRNAs previously found by our group to be deregulated in acute COVID-19 patients [23,24,25]. We found that LEF1-AS1 levels were significantly reduced in the PBMCs of patients with MPSs compared to those without MPSs.

Data indicate the specificity and the potential relevance of LEF1-AS1 deregulation. Indeed, while other lncRNAs, such as HCG18 or lncCEACAM21, were not modulated, all LEF1-AS1 isoforms tested were similarly deregulated in MPS patients. Moreover, when grouping patients for the persistence of MNSs, no significant changes were observed: the physio-pathological reasons for this difference were not investigated, but it may indicate the implication of LEF1-AS1 in MPSs. Accordingly, we observed a positive correlation between LEF1-AS1 and LEF1 and an inverse correlation between LEF1-AS1 isoforms and LEF1 with relevant clinical parameters.

LEF1-AS1-202 and -207 expression levels showed a weak but significant negative correlation with the age of long COVID patients, in line with the evidence indicating age as a risk factor for long COVID [10,30]. Moreover, the fact that lncRNAs were modulated only in patients with MPSs and not in patients with MNSs, and not in patients with MNSs but in no-MNS patients, suggests that age is not the only factor driving lncRNA dysregulation. Despite monocytes and eosinophil levels not changing significantly in MPS patients, they were negatively correlated with LEF1-AS1 and LEF1 expression levels, possibly indicating a weak but potentially relevant pro-inflammatory condition. This correlation is in accordance with evidence that suggest the persistence of monocyte activation and inflammation in long COVID. Accordingly, it has been shown that acute COVID-19 affects the relative proportions of monocyte subsets, leading to the activation of classical monocytes, and of pro-inflammatory cytokine production that persists at later stages of the disease [31]. Moreover, neutrophil and eosinophil responses remain abnormal for several months after the acute COVID-19 phase [32].

LEF1-AS1 is an antisense RNA to the lymphoid enhancer binding factor 1 (LEF1) gene, which encodes a transcription factor expressed mainly, but not exclusively, in pre-B and T cells. This factor is involved in cell proliferation, the activation of genes in the Wnt/β-catenin pathway, and the regulation of systemic inflammation. In cancer cells, LEF1-AS1 has a pro-oncogenic role, accelerating tumorigenesis and supporting cell proliferation and invasion in glioma [33] and non-small-cell lung cancer [34]. In acute COVID-19, the retained intron isoform 202 of LEF1-AS1 was identified as a potential biomarker for both the outcome and severity of the disease [24,25], displaying lower levels in more severe and non-surviving COVID-19 patients. Notably, the potential of this LEF1-AS1 isoform as an in-hospital mortality biomarker was evaluated in 1286 COVID-19 patients, recruited from four cohorts across Europe and Canada, using a lncRNA-based machine learning model that incorporated age and LEF1-AS1 expression as features [25]. Additionally, in a study conducted by Vausort et al. [35], LEF1-AS1-202 expression levels were measured in 104 mild COVID-19 patients during the acute phase; the patients were then stratified for the presence or absence of long COVID symptoms reported at 12 months from the acute illness. LEF1-AS1-202 was found downregulated in patients who developed long COVID symptoms, suggesting a prognostic potential [35]. In addition, the positive correlation of LEF1-AS1-202 with naïve T cells and the negative correlation with effector memory T cells indicate that it may influence the differentiation process, potentially impacting the generation of functional memory CD8+ T cells. Building on these findings, the downregulation of LEF1-AS1 observed in our MPS patient subgroup may reflect an impaired immune reprogramming following SARS-CoV-2 infection. Given that CD8+ T cells play a central role in viral clearance and the resolution of inflammation, we hypothesize that lower LEF1-AS1 levels could contribute to suboptimal T cell memory formation or sustained immune dysregulation. This, in turn, may predispose individuals to persistent somatic symptoms characteristic of MPSs in long COVID. Accordingly, LEF1, together with TCF-1, is essential for the generation of memory precursor cells and functional memory CD8+ T cells, as their absence leads to reduced expansion capacity [36]. Since LEF1-AS1 may modulate LEF1 transcriptional activity, its downregulation might disrupt this regulatory axis, ultimately compromising long-term antiviral immunity.

In long COVID patients, no inverse correlation with lymphocytes was observed, implicating that the decreased levels of LEF1 and LEF1-AS1 in MPSs may not simply be attributable to lower lymphocyte levels.

While all LEF1-AS1 isoforms display lower expression in MPS patients, upon adjustment for age and smoking habits, only the dysregulation of LEF1-AS1 multiple isoforms remained significant, while the significance of LEF1 modulation was borderline. This result is not unexpected, given the limited sample size of our study, but underscores the usefulness of an isoform-level study to fully exploit the potential of noncoding RNAs as disease biomarkers. Notably, although LEF1-AS1 multiple isoforms retain statistical significance, the age-dependence observed in some findings suggests that LEF1-AS1 may not be a standalone biomarker. This highlights the need to interpret its modulation within a broader biological and clinical framework. Further studies are required to assess whether the analysis of larger cohorts may allow us to identify the independent association of additional LEF1-AS1 isoforms and to more precisely determine the influence of confounding factors, such as smoking and age. Moreover, the positive correlation between all the investigated isoforms of LEF1-AS1 and LEF1 supports the idea of the co-dependent regulation of these molecules, suggesting a potential common pathway influenced by long COVID.

Some studies reveal that LEF1-AS1 can interact with LEF1 through different mechanisms. In colorectal cancer, LEF1-AS1 recruits MLL1 histone methyltransferase to the promoter of LEF1, stimulates H3K4me3 methylation, and activates LEF1 transcription [37]. Unfortunately, this study did not distinguish between LEF1-AS1 isoforms, and since they have significantly different sequences, studying each individually is of great importance, as they may work through different mechanisms. Indeed, Lu et al. found that LEF1-AS1-207 sponges the RNA binding protein HNRNPL which, in turn, stabilizes LEF1 mRNA [38]. 

Our study has several limitations. First, this is a monocentric study, and our cohort only includes patients who required hospitalization during the acute phase of COVID-19. Thus, LEF1-AS1 and LEF1 levels may not display long-term alterations in the PBMCs of patients that were affected by milder symptoms during acute COVID-19. Indeed, to fully elucidate the biological mechanisms underlying long COVID, future studies should include both hospitalized and non-hospitalized patients, as each group may contribute distinct insights into disease progression and long-term outcomes. Likewise, also analyzing healthy individuals may allow us to gain knowledge on the basal expression levels of LEF1-AS1 and LEF1. Second, participants were asked to return to our hospital for the questionnaire and blood sample collection, possibly biasing enrolment: older individuals and subjects with more severe long COVID symptoms who were unable to visit the hospital independently could have been excluded. Of note, these older and more severe patients might be those displaying most pronounced lncRNA dysregulation. Third, physical symptoms and diseases recorded during hospitalization were obtained from patient files, providing reliable data. However, pre-existing symptoms related to MNSs, such as anxiety and depression, were collected partially from patient files and partially from follow-up interviews. This latter source might be biased due to the subjective interpretation and memory of the patients. Fourth, we based the classification of MPS and MNS patients on the questionnaire administered, without using instrumental or objective measures to validate the presence and severity of long COVID symptoms. Fifth, insufficient information about pharmacological treatment at follow up was available, and this criterion could not be included in the analysis. Sixth, no power analysis was performed prior to the initiation of the study, representing a limitation to its design and the interpretation of the results.

In conclusion, this study identified LEF1-AS1 as a promising potential biomarker for MPSs in long COVID, contributing to our understanding of the molecular mechanisms underlying a specific aspect of the condition. Further studies are needed to validate and expand these findings, analyzing larger patient cohorts, including non-hospitalized individuals, those with mild COVID-19 symptoms, and healthy controls. Nevertheless, our discoveries offer new perspectives on the diagnosis and management of long COVID.

## 4. Materials and Methods

### 4.1. Ethics Approval and Consent to Participate

The study was performed in full compliance with the Declaration of Helsinki. The experimental protocol of IRCCS Policlinico San Donato (protocol number 39/INT/2022, of 6 April 2022) was approved by the Institutional Ethics Committee of San Raffaele Hospital. Prior to enrolment, participants were requested to provide informed consent, as previously approved by the ethics committee.

### 4.2. Patient Selection and Sample Collection

Subjects hospitalized at IRCCS Policlinico San Donato due to acute COVID-19 between January 2021 and June 2022 constituted the eligible patient population for the “long COVID” protocol. This nested case–control study enrolled a total of 98 patients (Figure S7). Patients aged between 18 and 90 years old were contacted through a telephone call during the period between July 2022 and December 2022. Among the eligible population, a subset of subjects declined participation while the others agreed to come to the hospital for enrolment. The non-participating group had a median age of 79 years (*p* < 0.0001) and was sex-matched with the studied population. The protocol of the study included extracting pertinent data about COVID-19 hospitalization, the administration of the long COVID questionnaire validated by the Italian Ministry of Health Data [5] via in-person interviews conducted by a biologist and a medical doctor, and the collection of peripheral blood samples. Platelet-poor plasma and peripheral blood mononuclear cell (PBMC) samples were prepared according to internal Standard Operating Procedures by BioCor Biobank at IRCCS Policlinico San Donato, part of BBMRI-ERIC and BBMRI.it and operating according to national and international guidelines.

The questionnaire included items comprising a wide array of symptoms related to long COVID. For each symptom, patients were asked to specify whether it was still present at the time of the interview, had been present but was resolved, or was present intermittently. Based on symptoms not present before hospitalization for COVID-19 and still present at the time of the interview, patients were classified into two different groups, as previously described [5]. Those who exhibited at least one persistent symptom among fever, persistent cough, chronic fatigue, and shortness of breath were classified as patients affected by major physical symptoms (MPSs), while those who exhibited at least one persistent symptom among anxiety, depression, brain fog, and memory dysfunction were classified as patients with major neuropsychological symptoms (MNSs). Participant data were collected at the time of the interview using the Research Electronic Data Capture (RedCAP) platform.

### 4.3. RNA Isolation and RT-qPCR Quantification

RNA isolation from PBMC samples was carried out using Trizol RNA Extraction reagent (#FS-881-200, Società Italiana Chimici, Rome, Italy) as previously described [39]. Before reverse transcription, 1 µg of RNA was digested with 1 U of DNase I (#18068-015, ThermoFisher Scientific, Massachusetts, USA) according to the manufacturer’s protocol. The digestion mixture was incubated at 37 °C for 30 min, and then the reaction was inactivated by adding 50 mM EDTA and incubating the samples at 65 °C for 10 min. Reverse transcriptase reactions were then conducted in the presence (RT+) or in the absence (RT−) of the enzyme, followed by PCR amplification. RT− samples did not show any amplification signal. lncRNA levels were measured with GoTaq^®^ qPCR Master Mix (#A6002, Promega, Wisconsin, USA) and were normalized to UBC levels. Primer sequences are listed in Table S3; alignment was performed using GRCh38/hg38 Ensembl Genome Browser. The raw Cts of investigated transcripts are listed in Table S4. The size of the LEF1-AS1 isoforms and LEF1 amplification products were visualized using 2% agarose gel electrophoresis (Figures S3 and S8). RNA isolation from platelet-poor plasma samples was performed with NucleoSpin miRNA Plasma (#740981.50, MACHEREY–NAGEL, Düren, Germany) following the manufacturer’s instructions. miRNA expression levels were investigated as previously described [23]. Specific miRNA primers were provided by ThermoFisher Scientific, and the expression values were normalized to U6 levels. All RNA species were quantified using the CFX Opus 96 Real-time PCR System (BIO-RAD, Hercules, CA, USA), and relative expression was calculated using the comparative Ct (Delta Delta Ct) method [40].

### 4.4. Statistical Analysis

Continuous variables are shown as means ±  standard deviation (SD). Group-wise comparisons were conducted using either the Mann–Whitney test or unpaired *t*-test, as appropriate. All statistical tests were two-sided, with the significance level set at *p* ≤ 0.05.

Descriptive statistics were expressed with counts and percentages for categorical variables and median values with the interquartile range (IQR) for continuous variables.

The association between MPSs and MNSs and baseline demographic or clinical variables was investigated with the Chi-squared test or Fisher’s exact test for categorical variables; continuous variables were compared by the nonparametric Kruskal–Wallis test for non-normally distributed data.

Correlations between continuous variables were evaluated according to Pearson R or Spearman Rho, depending on the distribution.

The relation between the baseline concentrations of the biomarkers and the outcomes was first tested with univariate logistic regression models. Multi-variable logistic regression was used to identify factors at admission that were independent predictors of MPSs. All of the variables associated with the outcome in the univariate analysis (*p* < 0.01) were included in the multiple logistic regression models. Odds ratios (ORs) with their corresponding 95% CIs were calculated, and *p* values were considered statistically significant if they were less than 0.05.

All statistical analyses were performed with SAS software, version 9.4 (SAS Institute, Inc., Cary, NC, USA), and GraphPad Prism 8.3.0 (GraphPad Software, Inc., La Jolla, CA, USA).

## Figures and Tables

**Table 1:** Patient characteristics at hospital admission and during acute COVID-19 disease.

	ALL (N = 98)	MPS (N = 48)	NO MPS (N = 50)	Odds Ratio (95% CI)	*p* Value	MNS (N = 39)	NO MNS (N = 59)	Odd Ratio (95% CI)	*p* Value
Age, median (range)	66 (55–74)	71 (60–77)	58 (49–67)	1.06 (1.02–1.10)	0.0011	71 (61–78)	59 (52–71)	1.05 (1.02–1.09)	0.0047
Gender, male, *n* (%)	67 (68.37)	29 (60.42)	38 (76.00)	2.08 (0.87–4.95)	0.0999	21 (53.85)	46 (77.97)	3.03 (1.26–7.32)	0.0135
**Medical history/** **comorbidities, *n* (%)**									
Current smoker	5 (5.10)	2 (4.17)	3 (6.00)	0.68 (0.10–4.27)	0.6817	1 (2.56)	4 (6.78)	0.36 (0.04–3.37)	0.3716
Former smoker	43 (43.88)	28 (58.33)	15 (30.00)	3.27 (1.42–7.52)	0.0054	20 (51.28)	23 (38.98)	1.65 (0.73–3.73)	0.2312
Hypertension	46 (46.94)	28 (58.33)	19 (36.00)	2.49 (1.10–5.62)	0.0281	21 (53.85)	25 (42.37)	1.58 (0.70–3.58)	0.2665
Diabetes	16 (16.33)	10 (20.85)	6 (12.00)	1.93 (0.0.64–5.81)	0.2420	11 (28.21)	5 (8.47)	4.24 (1.34–13.42)	0.0139
Obesity	24 (24.49)	13 (27.08)	11 (22.00)	1.32 (0.52–3.32)	0.5596	11 (28.21)	13 (22.03)	1.39 (0.55–3.52)	0.4877
Ischemic cardiomyopathy	17 (17.35)	8 (16.67)	9 (18.00)	0.91 (0.32–2.60)	0.8618	9 (23.08)	8 (13.56)	1.91 (0.67–5.49)	0.2278
Chronic heart failure	7 (7.14)	4 (8.33)	3 (6.00)	1.42 (0.30–6.73)	0.6552	3 (7.69)	4 (6.78)	1.15 (0.24–5.43)	0.8637
Atrial fibrillation	9 (9.18)	8 (16.67)	1 (2.00)	9.80 (1.18–81.68)	0.0349	5 (12.82)	4 (6.78)	2.02 (0.51–8.06)	0.3182
Left ventricular disfunction	1 (1.02)	0 (0.00)	1 (2.00)	-	-	0 (0.00)	1 (1.69)	-	-
BPCO	5 (5.10)	3 (6.25)	2 (4.00)	1.60 (0.26–10.03)	0.6156	2 (5.13)	3 (5.08)	1.00 (0.16–6.33)	0.9924
Asthma	4 (4.08)	3 (6.25)	1 (2.00)	3.28 (0.33–32.55)	0.3129	2 (5.13)	2 (3.39)	1.54 (0.21–11.42)	0.6724
Cancer	9 (9.18)	3 (6.25)	6 (12.00)	0.49 (0.12–2.08)	0.3324	6 (15.38)	3 (5.08)	3.39 (0.79–14.48)	0.0988
Pre-existing stroke	5 (5.10)	3 (6.25)	2 (4.00)	1.60 (0.26–10.02)	0.6256	1 (2.56)	4 (6.78)	0.36 (0.04–3.37)	0.3716
Chronic neurological disorders	3 (3.06)	3 (6.25)	0 (0.00)	-	-	1 (2.56)	2 (3.39)	0.75 (0.07–8.56)	0.8169
Chronic kidney disorders	7 (7.14)	3 (6.25)	4 (8.00)	0.77 (0.16–3.62)	0.7373	3 (7.69)	4 (6.78)	1.15 (0.24–5.43)	0.8637
Liver disorders	3 (3.06)	1 (2.08)	2 (4.00)	0.51 (0.05–5.82)	0.5884	2 (5.13)	1 (1.69)	3.14 (0.27–35.81)	0.3578
Chronic gut inflammation	3 (3.06)	3 (6.25)	0 (0.00)	-	-	2 (5.13)	1 (1.69)	3.14 (0.28–35.81)	0.3578
Anxiety	11 (11.22)	8 (16.67)	3 (6.00)	3.13 (0.78–12.61)	0.1079	8 (20.51)	3 (5.08)	4.82 (1.91–19.49)	0.0275
Depression	11 (11.22)	7 (14.59)	4 (8.00)	1.96 (0.54–7.19)	0.3086	8 (20.51)	3 (5.08)	4.82 (1.91–19.49)	0.0275
**COVID-19 disease**									
Days of hospitalization, median (range)	14 (10–22)	14 (10–22)	15 (10–25)	0.99 (0.96–1.02)	0.4097	14 (9–23)	14 (10–22)	1.01 (0.98–1.04)	0.7099
No oxygen therapy, *n* (%)	17 (17)	3 (6)	14 (28)	0.17 (0.05–0.66)	0.01	5 (12)	12 (20)	0.56 (0.18–1.75)	ns
Oxygen therapy, *n* (%)	51 (52.04)	27 (56.25)	24 (48.00)	1.39 (0.63–3.07)	0.4143	21 (53.85)	30 (50.85)	1.13 (0.50–2.54)	0.7712
CPAP therapy, *n* (%)	27 (27.55)	17 (35.42)	10 (20.00)	2.19 (0.88–5.46)	0.0911	11 (28.21)	16 (27.12)	1.06 (0.43–2.61)	0.9060
Admission to ICU, *n* (%)	3 (3.06)	1 (2.08)	2 (4.00)	0.51(0.05–5.82)	0.5884	1 (2.56)	2 (3.39)	0.75 (0.07–8.56)	0.8169

MPS, major physical symptom (indicated in the table with a yellow background); MNS, major neuropsychological symptom (indicated in the table with a green background).

**Table 2:** Symptoms developed after hospital discharge and still present at the moment of the interview.

	ALL (N = 98)	MPS (N = 48)	NO MPS (N = 50)	*p* Value	MNS (N = 39)	NO MNS (N = 59)	*p* Value
**Months from COVID-19 disease, median (range)**	18 (16–19)	18 (17–19)	17 (12–19)	0.2356	18 (17–19)	18 (16–19)	0.8543
**Symptoms developed after COVID-19 disease, number of patients (%)**							
Abdominal pain/diarrhea	1 (1.02)	1 (2.08)	0 (0.00)	0.4898 ^#^	1 (2.56)	0 (0.00)	0.3980 ^#^
Anxiety	15 (15.31)	13 (27.08)	2 (4.00)	0.0015	15 (38.46)	0 (0.00)	<0.0001
Arthralgia	26 (26.53)	20 (41.67)	6 (12.00)	0.0009	17 (43.59)	9 (15.25)	0.0019
Brain fog	9 (9.18)	6 (12.50)	3 (6.00)	0.3128 ^#^	9 (23.08)	0 (0.00)	0.0001 ^#^
Chest pain	2 (2.04)	2 (4.17)	0 (0.00)	0.2373 ^#^	0 (0.00)	2 (3.39)	0.5159 ^#^
Decreased appetite	4 (4.08)	4 (8.33)	0 (0.00)	0.0539 ^#^	3 (7.69)	1 (1.69)	0.2980 ^#^
Delirium	4 (4.08)	4 (8.33)	0 (0.00)	0.0539	3 (7.69)	1(1.69)	0.2980 ^#^
Depression	15 (15.31)	12 (25.00)	3 (6.00)	0.0090	15 (38.46)	0 (0.00)	<0.0001
Difficulties in concentrating	17 (17.35)	13 (27.08)	4 (8.00)	0.0126	16 (41.03)	1 (1.69)	<0.0001
Dizziness and balance disorders	23 (23.47)	21 (43.75)	2 (4.00)	<0.0001	16 (41.03)	7 (11.86)	0.0009
Dyspnoea/shortness of breath	34 (34.69)	34 (70.83)	0 (0.00)	<0.0001	20 (51.28)	14 (23.73)	0.0050
Headache	6 (6.12)	4 (8.33)	2 (4.00)	0.412	3 (7.69)	3 (5.08)	0.6796 ^#^
High temperature	0 (0.00)	0 (0.00)	0 (0.00)	-	0 (0.00)	0 (0.00)	-
Memory dysfunction	30 (30.61)	22 (45.83)	8 (16.00)	0.0014	30 (76.92)	0 (0.00)	<0.0001
Myalgia	19 (19.39)	14 (29.17)	5 (10.00)	0.0164	11 (28.21)	8 (13.56)	0.0726
Nausea/vomiting	1 (1.02)	1 (2.08)	0 (0.00)	0.4898 ^#^	1 (2.56)	0 (0.00)	0.3980 ^#^
Palpitations/tachycardia	3 (3.06)	3 (6.25)	0 (0.00)	0.1137 ^#^	3 (7.69)	0 (0.00)	0.0601 ^#^
Persistent cough	10 (10.20)	10 (20.83)	0 (0.00)	0.0005 ^#^	3 (7.69)	7 (11.86)	0.7354 ^#^
Persistent fatigue	36 (36.73)	38 (75.00)	0 (0.00)	<0.0001	22 (56.41)	14 (23.73)	0.0010
Sleep disorders	22 (22.45)	18 (37.5)	4 (8.00)	0.0005	15 (38.46)	7 (11.86)	0.0020
Tingling and numbness	13 (13.27)	11 (22.92)	2 (4.00)	0.0058	10 (25.64)	3 (5.08)	0.0033
Weight loss	1 (1.02)	1 (2.08)	0 (0.00)	0.4898 ^#^	0 (0.00)	1 (1.69)	1.000 ^#^

MPS, major physical symptom (indicated in the table with a yellow background); MNS, major neuropsychological symptom (indicated in the table with a green background). ^#^ Fisher test.

## Data Availability

Data that support the findings of this study, which are not publicly available due to privacy or ethical restrictions, can be obtained upon request from the corresponding author.

## Supplementary Materials

The following supporting information can be downloaded at: https://www.mdpi.com/article/10.3390/ijms26104806/s1.
