# Supplementary material for: LEF1-AS1 Deregulation in the Peripheral Blood of Patients with Persistent Post-COVID Symptoms
Source: Int J Mol Sci. 2025 May 17;26(10):4806. doi: 10.3390/ijms26104806 (PMC12112689; doi:10.3390/ijms26104806)
Supplement: Supplementary file 1 [file ijms-26-04806-s001.zip › ijms-3604011-supplementary.pptx]

## Slide 1
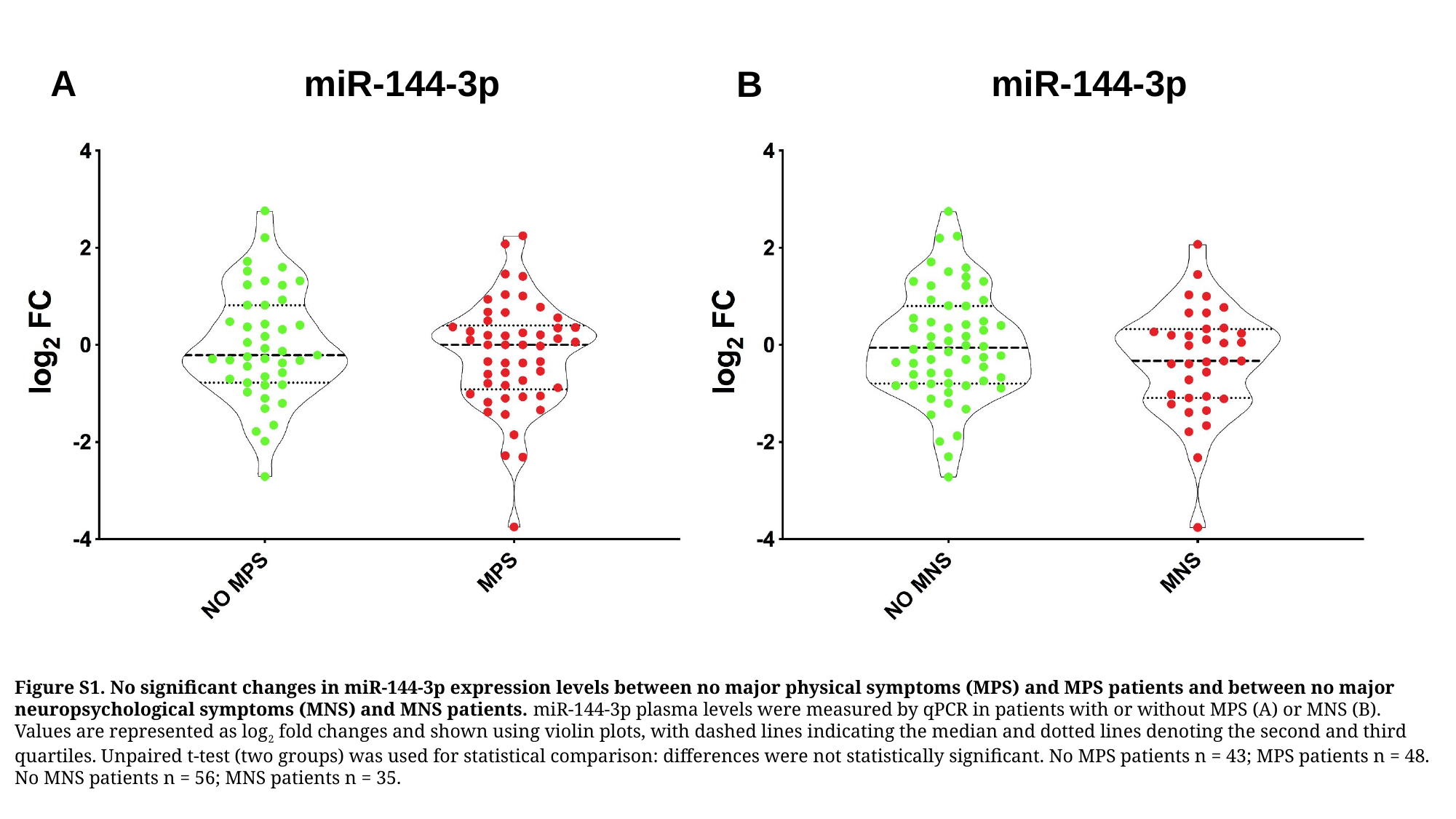

miR-144-3p
miR-144-3p
A
B
Figure S1. No significant changes in miR-144-3p expression levels between no major physical symptoms (MPS) and MPS patients and between no major neuropsychological symptoms (MNS) and MNS patients. miR-144-3p plasma levels were measured by qPCR in patients with or without MPS (A) or MNS (B). Values are represented as log2 fold changes and shown using violin plots, with dashed lines indicating the median and dotted lines denoting the second and third quartiles. Unpaired t-test (two groups) was used for statistical comparison: differences were not statistically significant. No MPS patients n = 43; MPS patients n = 48. No MNS patients n = 56; MNS patients n = 35.

## Slide 2
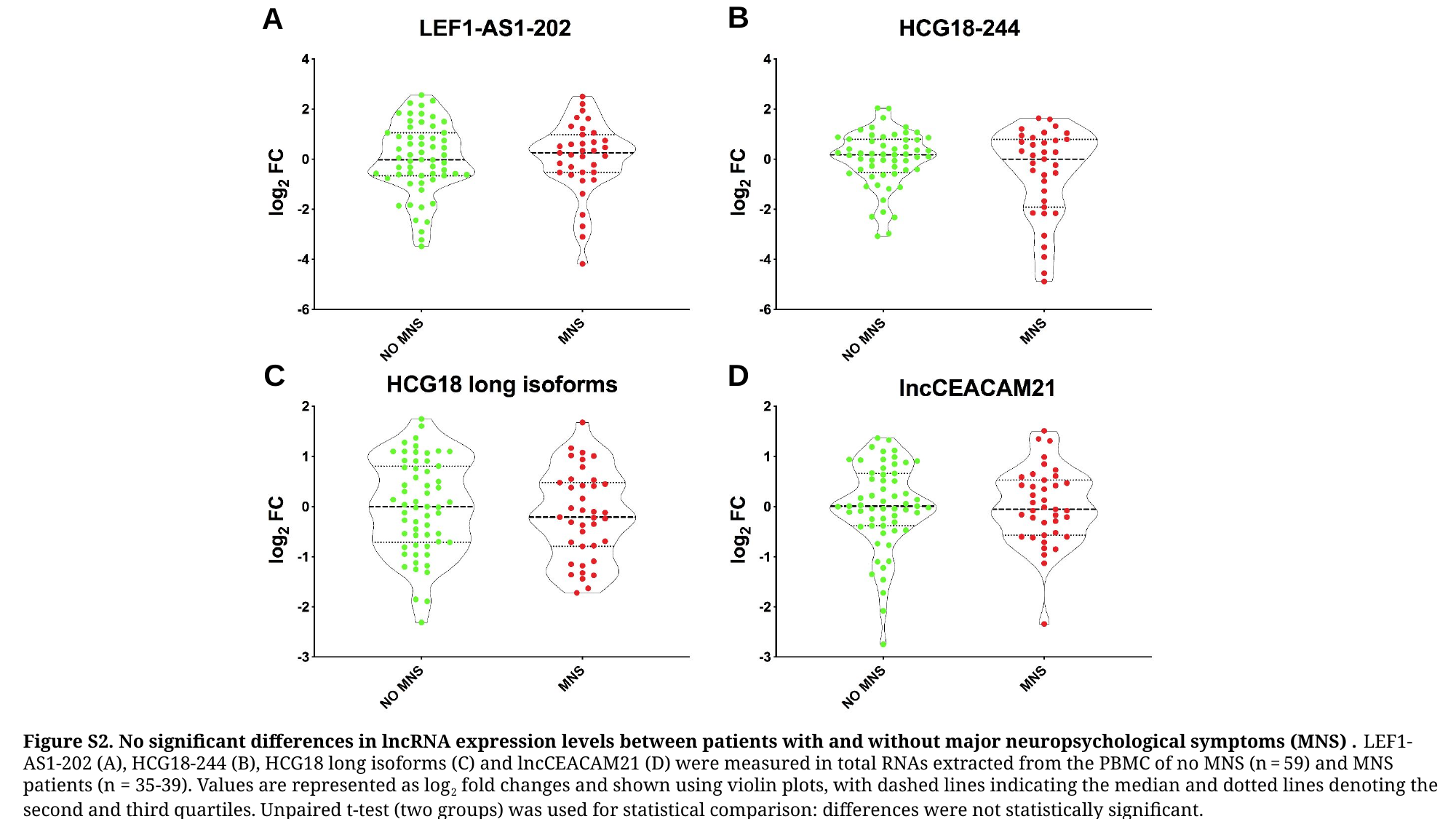

B
A
C
D
Figure S2. No significant differences in lncRNA expression levels between patients with and without major neuropsychological symptoms (MNS) . LEF1-AS1-202 (A), HCG18-244 (B), HCG18 long isoforms (C) and lncCEACAM21 (D) were measured in total RNAs extracted from the PBMC of no MNS (n = 59) and MNS patients (n = 35-39). Values are represented as log2 fold changes and shown using violin plots, with dashed lines indicating the median and dotted lines denoting the second and third quartiles. Unpaired t-test (two groups) was used for statistical comparison: differences were not statistically significant.

## Slide 3
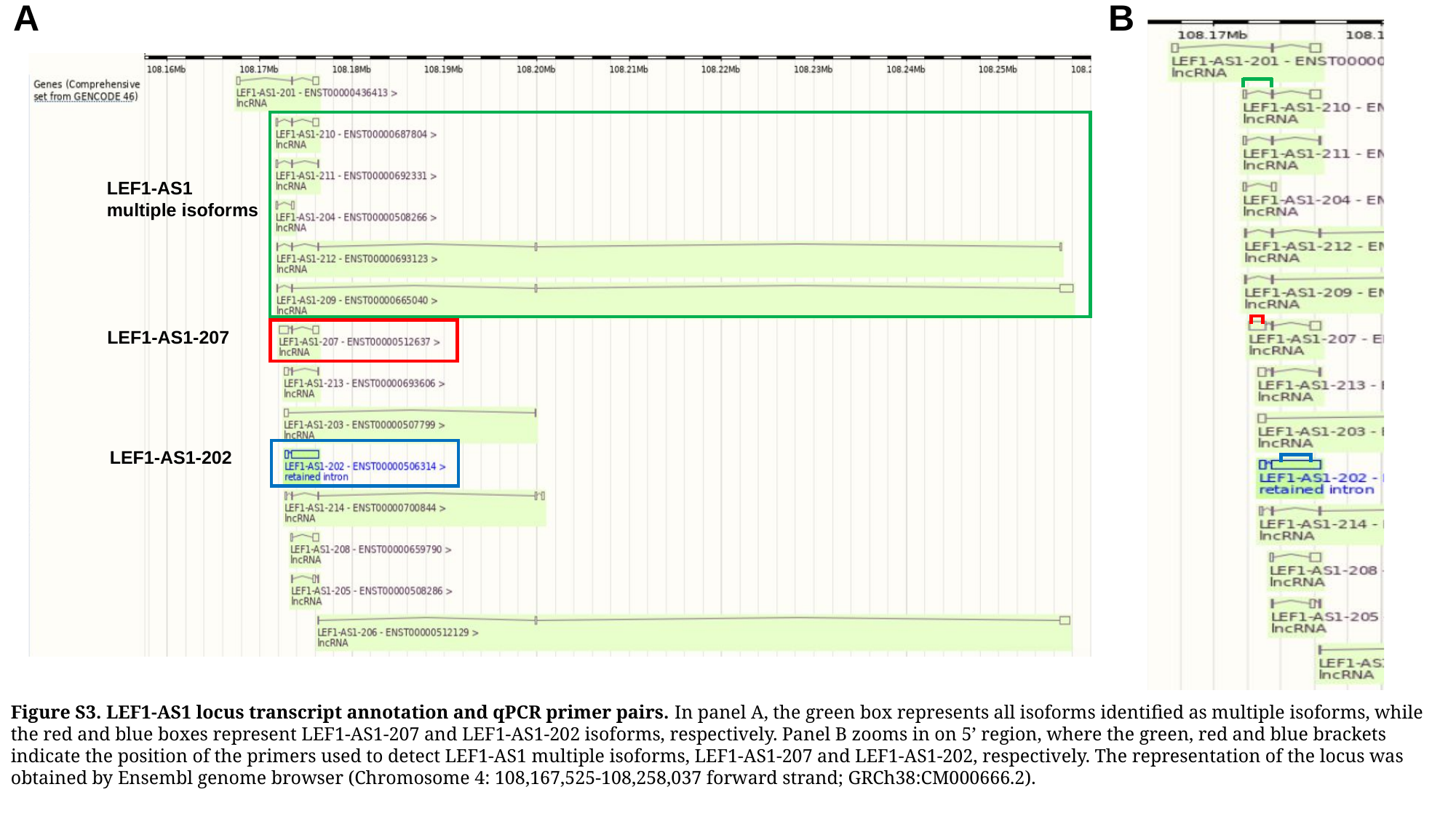

A
B
LEF1-AS1
multiple isoforms
LEF1-AS1-207
LEF1-AS1-202
Figure S3. LEF1-AS1 locus transcript annotation and qPCR primer pairs. In panel A, the green box represents all isoforms identified as multiple isoforms, while the red and blue boxes represent LEF1-AS1-207 and LEF1-AS1-202 isoforms, respectively. Panel B zooms in on 5’ region, where the green, red and blue brackets indicate the position of the primers used to detect LEF1-AS1 multiple isoforms, LEF1-AS1-207 and LEF1-AS1-202, respectively. The representation of the locus was obtained by Ensembl genome browser (Chromosome 4: 108,167,525-108,258,037 forward strand; GRCh38:CM000666.2).

## Slide 4
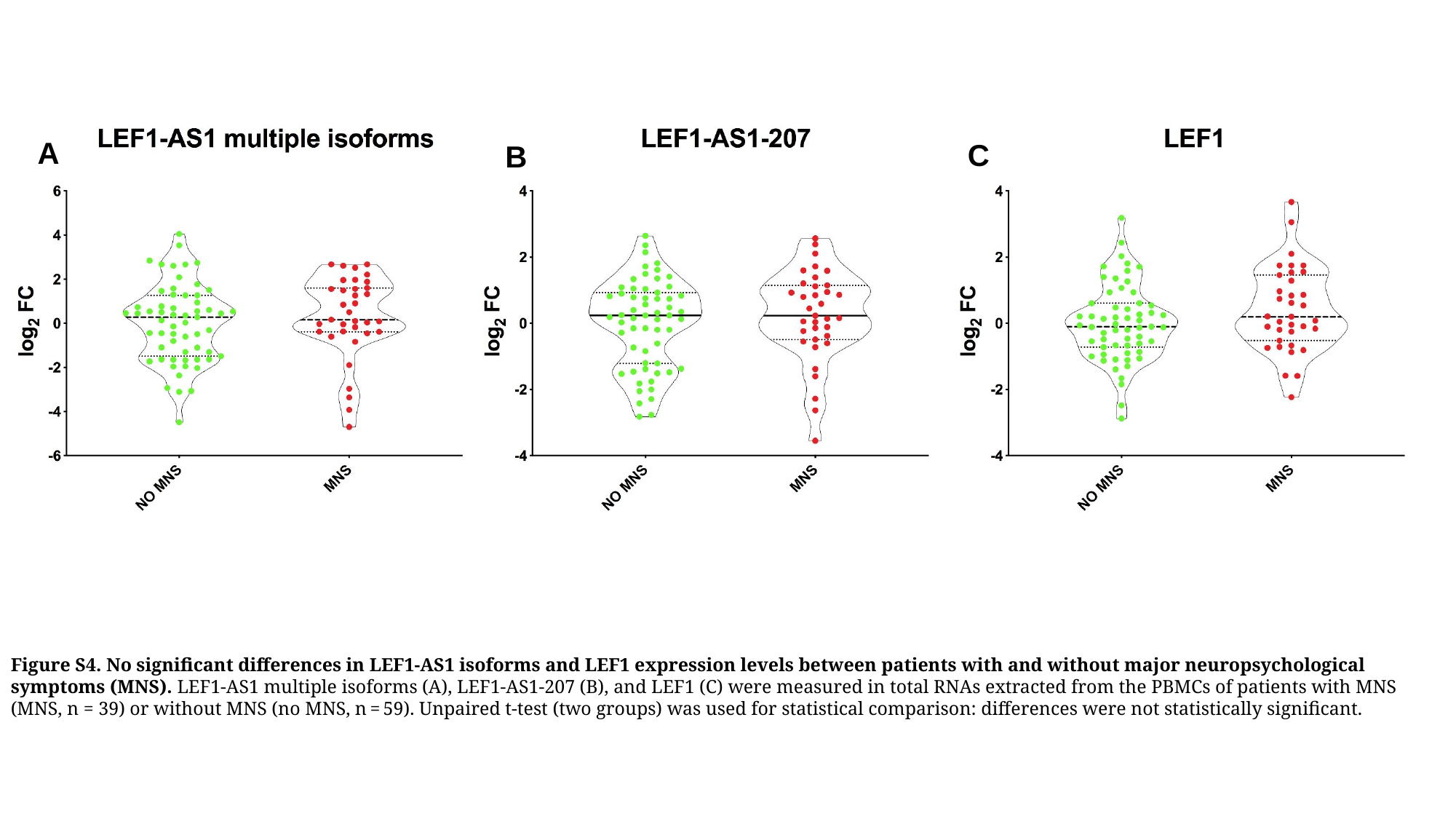

A
C
B
Figure S4. No significant differences in LEF1-AS1 isoforms and LEF1 expression levels between patients with and without major neuropsychological symptoms (MNS). LEF1-AS1 multiple isoforms (A), LEF1-AS1-207 (B), and LEF1 (C) were measured in total RNAs extracted from the PBMCs of patients with MNS (MNS, n = 39) or without MNS (no MNS, n = 59). Unpaired t-test (two groups) was used for statistical comparison: differences were not statistically significant.

## Slide 5
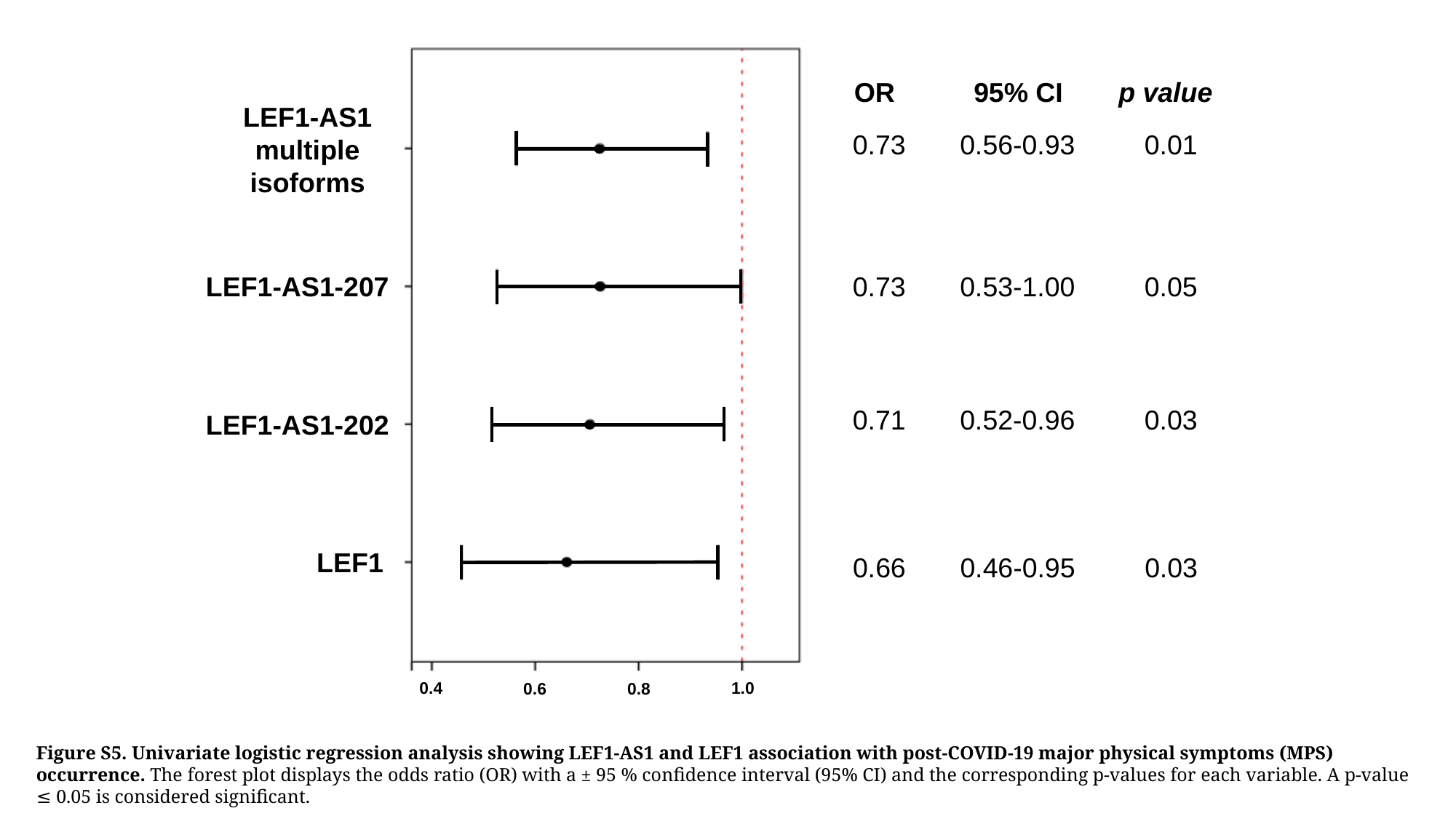

LEF1-AS1 multiple isoforms
LEF1-AS1-207
LEF1-AS1-202
LEF1
0.4
1.0
0.6
0.8
OR
95% CI
p value
0.73
0.56-0.93
0.01
0.73
0.53-1.00
0.05
0.71
0.52-0.96
0.03
0.66
0.46-0.95
0.03
Figure S5. Univariate logistic regression analysis showing LEF1-AS1 and LEF1 association with post-COVID-19 major physical symptoms (MPS) occurrence. The forest plot displays the odds ratio (OR) with a ± 95 % confidence interval (95% CI) and the corresponding p-values for each variable. A p-value ≤ 0.05 is considered significant.

## Slide 6
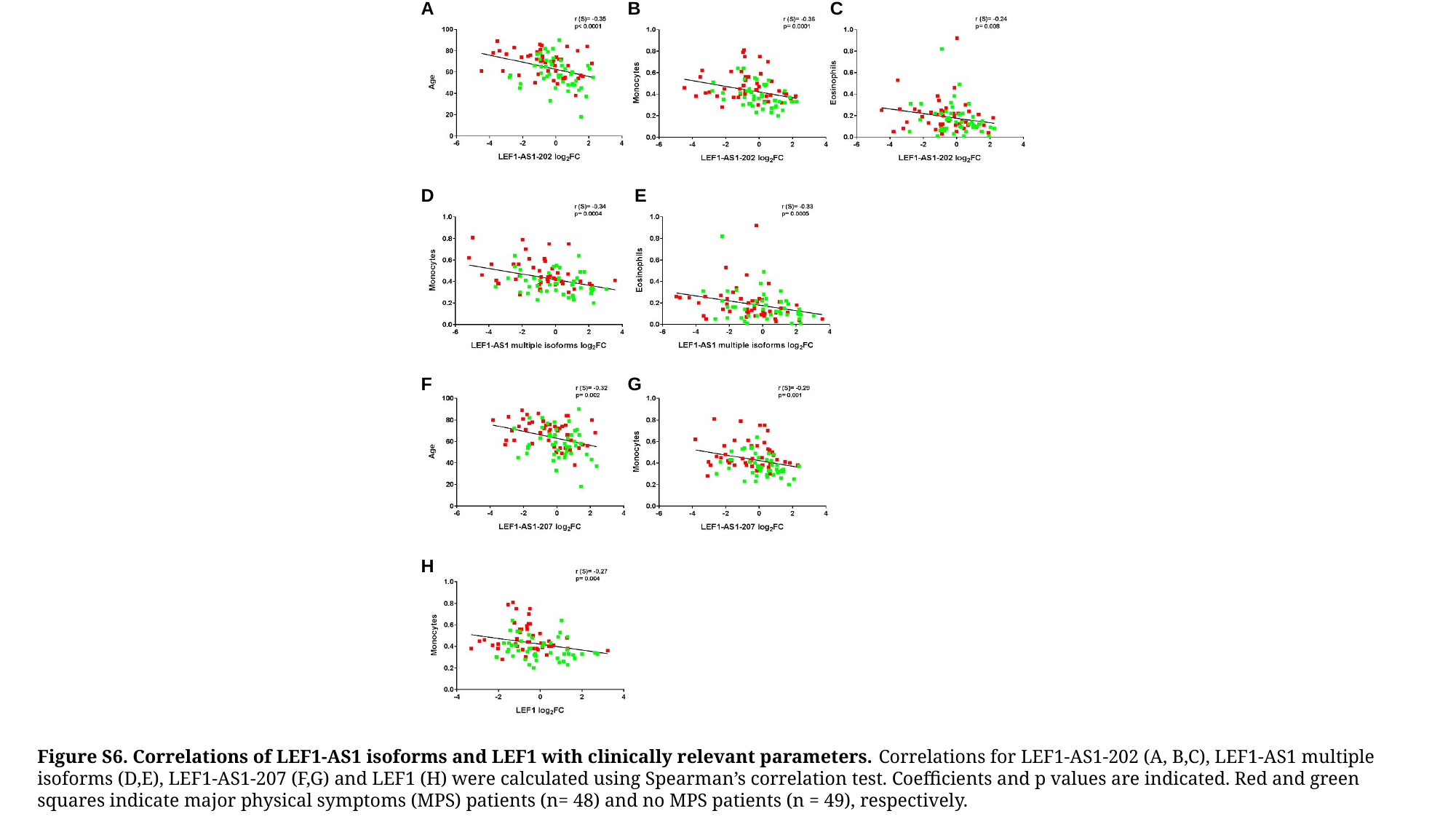

A
C
B
E
D
G
F
H
Figure S6. Correlations of LEF1-AS1 isoforms and LEF1 with clinically relevant parameters. Correlations for LEF1-AS1-202 (A, B,C), LEF1-AS1 multiple isoforms (D,E), LEF1-AS1-207 (F,G) and LEF1 (H) were calculated using Spearman’s correlation test. Coefficients and p values are indicated. Red and green squares indicate major physical symptoms (MPS) patients (n= 48) and no MPS patients (n = 49), respectively.

## Slide 7
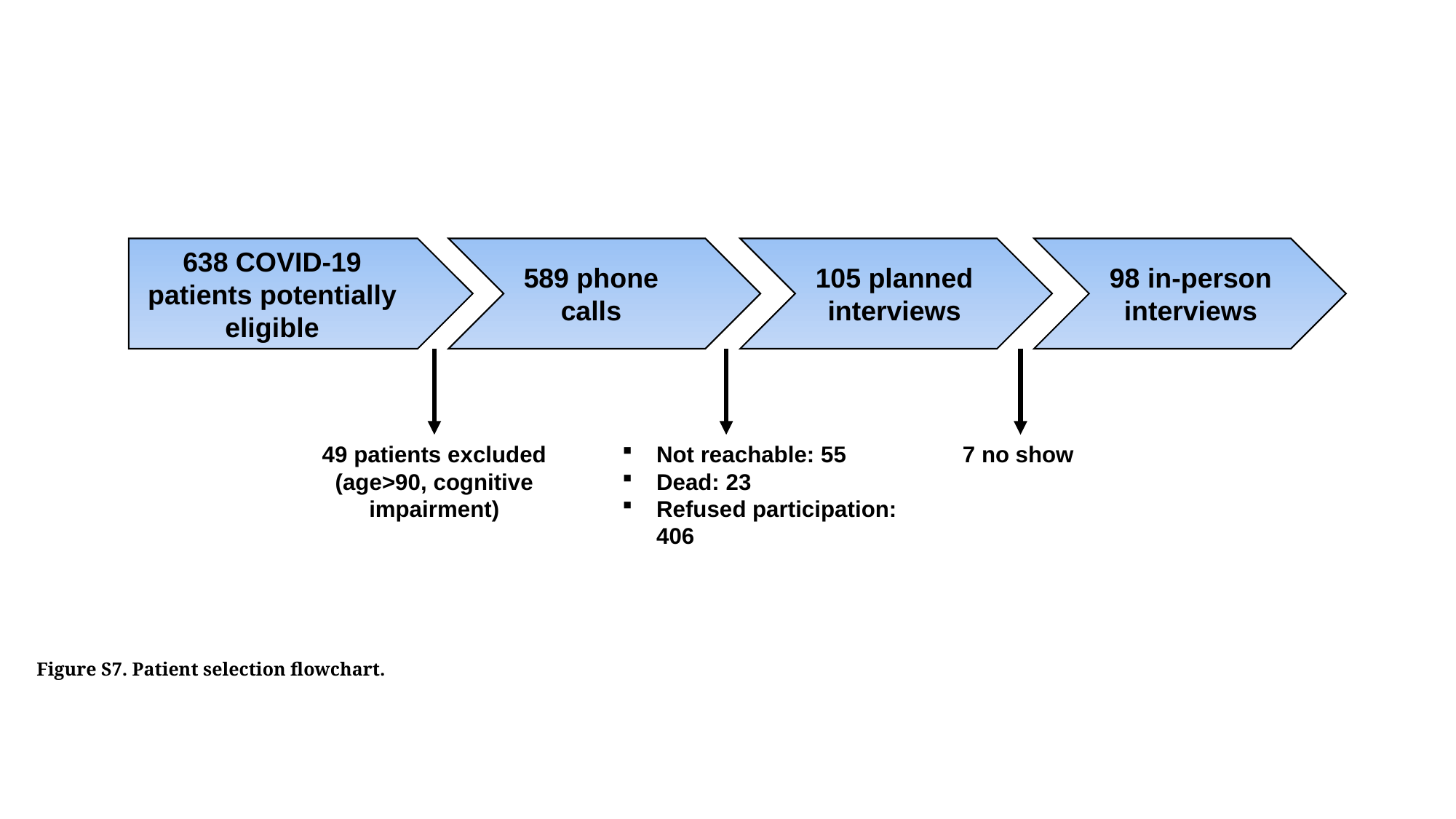

638 COVID-19 patients potentially eligible
105 planned
interviews
98 in-person interviews
589 phone
calls
7 no show
49 patients excluded (age>90, cognitive impairment)
Not reachable: 55
Dead: 23
Refused participation: 406
Figure S7. Patient selection flowchart.

## Slide 8
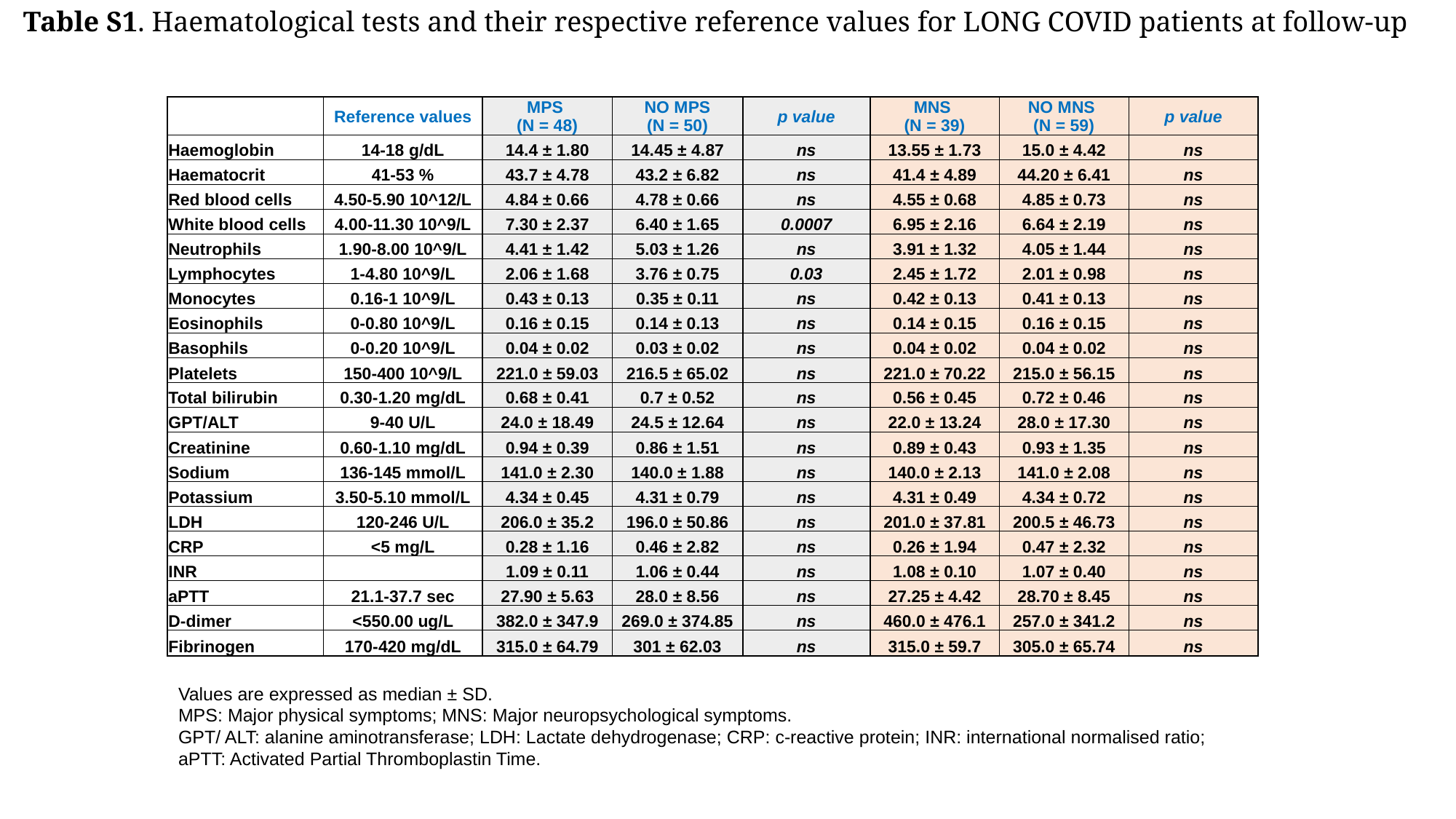

Table S1. Haematological tests and their respective reference values for LONG COVID patients at follow-up
| | Reference values | MPS (N = 48) | NO MPS (N = 50) | p value | MNS (N = 39) | NO MNS (N = 59) | p value |
| --- | --- | --- | --- | --- | --- | --- | --- |
| Haemoglobin | 14-18 g/dL | 14.4 ± 1.80 | 14.45 ± 4.87 | ns | 13.55 ± 1.73 | 15.0 ± 4.42 | ns |
| Haematocrit | 41-53 % | 43.7 ± 4.78 | 43.2 ± 6.82 | ns | 41.4 ± 4.89 | 44.20 ± 6.41 | ns |
| Red blood cells | 4.50-5.90 10^12/L | 4.84 ± 0.66 | 4.78 ± 0.66 | ns | 4.55 ± 0.68 | 4.85 ± 0.73 | ns |
| White blood cells | 4.00-11.30 10^9/L | 7.30 ± 2.37 | 6.40 ± 1.65 | 0.0007 | 6.95 ± 2.16 | 6.64 ± 2.19 | ns |
| Neutrophils | 1.90-8.00 10^9/L | 4.41 ± 1.42 | 5.03 ± 1.26 | ns | 3.91 ± 1.32 | 4.05 ± 1.44 | ns |
| Lymphocytes | 1-4.80 10^9/L | 2.06 ± 1.68 | 3.76 ± 0.75 | 0.03 | 2.45 ± 1.72 | 2.01 ± 0.98 | ns |
| Monocytes | 0.16-1 10^9/L | 0.43 ± 0.13 | 0.35 ± 0.11 | ns | 0.42 ± 0.13 | 0.41 ± 0.13 | ns |
| Eosinophils | 0-0.80 10^9/L | 0.16 ± 0.15 | 0.14 ± 0.13 | ns | 0.14 ± 0.15 | 0.16 ± 0.15 | ns |
| Basophils | 0-0.20 10^9/L | 0.04 ± 0.02 | 0.03 ± 0.02 | ns | 0.04 ± 0.02 | 0.04 ± 0.02 | ns |
| Platelets | 150-400 10^9/L | 221.0 ± 59.03 | 216.5 ± 65.02 | ns | 221.0 ± 70.22 | 215.0 ± 56.15 | ns |
| Total bilirubin | 0.30-1.20 mg/dL | 0.68 ± 0.41 | 0.7 ± 0.52 | ns | 0.56 ± 0.45 | 0.72 ± 0.46 | ns |
| GPT/ALT | 9-40 U/L | 24.0 ± 18.49 | 24.5 ± 12.64 | ns | 22.0 ± 13.24 | 28.0 ± 17.30 | ns |
| Creatinine | 0.60-1.10 mg/dL | 0.94 ± 0.39 | 0.86 ± 1.51 | ns | 0.89 ± 0.43 | 0.93 ± 1.35 | ns |
| Sodium | 136-145 mmol/L | 141.0 ± 2.30 | 140.0 ± 1.88 | ns | 140.0 ± 2.13 | 141.0 ± 2.08 | ns |
| Potassium | 3.50-5.10 mmol/L | 4.34 ± 0.45 | 4.31 ± 0.79 | ns | 4.31 ± 0.49 | 4.34 ± 0.72 | ns |
| LDH | 120-246 U/L | 206.0 ± 35.2 | 196.0 ± 50.86 | ns | 201.0 ± 37.81 | 200.5 ± 46.73 | ns |
| CRP | <5 mg/L | 0.28 ± 1.16 | 0.46 ± 2.82 | ns | 0.26 ± 1.94 | 0.47 ± 2.32 | ns |
| INR | | 1.09 ± 0.11 | 1.06 ± 0.44 | ns | 1.08 ± 0.10 | 1.07 ± 0.40 | ns |
| aPTT | 21.1-37.7 sec | 27.90 ± 5.63 | 28.0 ± 8.56 | ns | 27.25 ± 4.42 | 28.70 ± 8.45 | ns |
| D-dimer | <550.00 ug/L | 382.0 ± 347.9 | 269.0 ± 374.85 | ns | 460.0 ± 476.1 | 257.0 ± 341.2 | ns |
| Fibrinogen | 170-420 mg/dL | 315.0 ± 64.79 | 301 ± 62.03 | ns | 315.0 ± 59.7 | 305.0 ± 65.74 | ns |
Values are expressed as median ± SD.
MPS: Major physical symptoms; MNS: Major neuropsychological symptoms.
GPT/ ALT: alanine aminotransferase; LDH: Lactate dehydrogenase; CRP: c-reactive protein; INR: international normalised ratio; aPTT: Activated Partial Thromboplastin Time.

## Slide 9
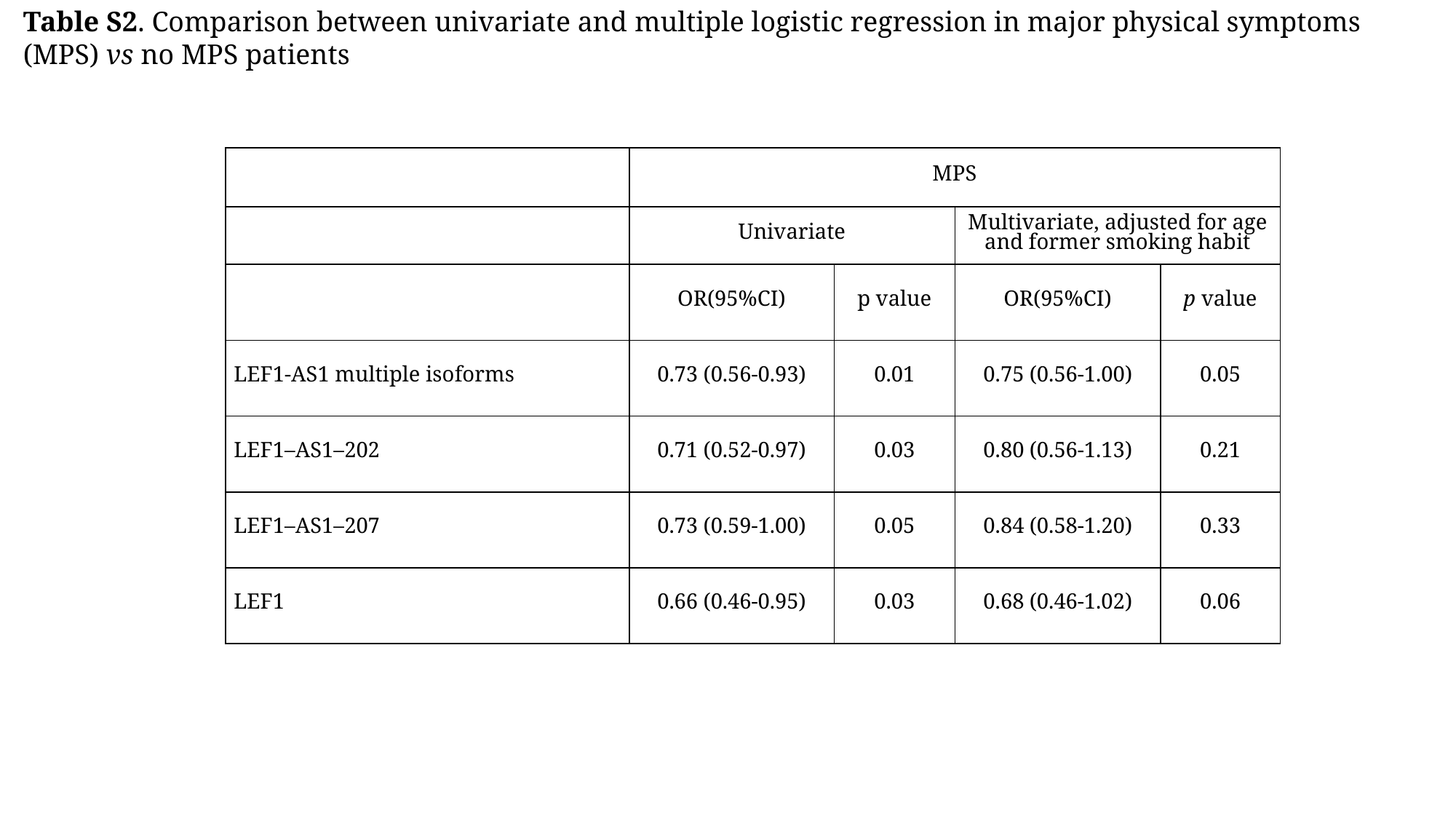

Table S2. Comparison between univariate and multiple logistic regression in major physical symptoms (MPS) vs no MPS patients
| | MPS | | | |
| --- | --- | --- | --- | --- |
| | Univariate | | Multivariate, adjusted for age and former smoking habit | |
| | OR(95%CI) | p value | OR(95%CI) | p value |
| LEF1-AS1 multiple isoforms | 0.73 (0.56-0.93) | 0.01 | 0.75 (0.56-1.00) | 0.05 |
| LEF1–AS1–202 | 0.71 (0.52-0.97) | 0.03 | 0.80 (0.56-1.13) | 0.21 |
| LEF1–AS1–207 | 0.73 (0.59-1.00) | 0.05 | 0.84 (0.58-1.20) | 0.33 |
| LEF1 | 0.66 (0.46-0.95) | 0.03 | 0.68 (0.46-1.02) | 0.06 |

## Slide 10
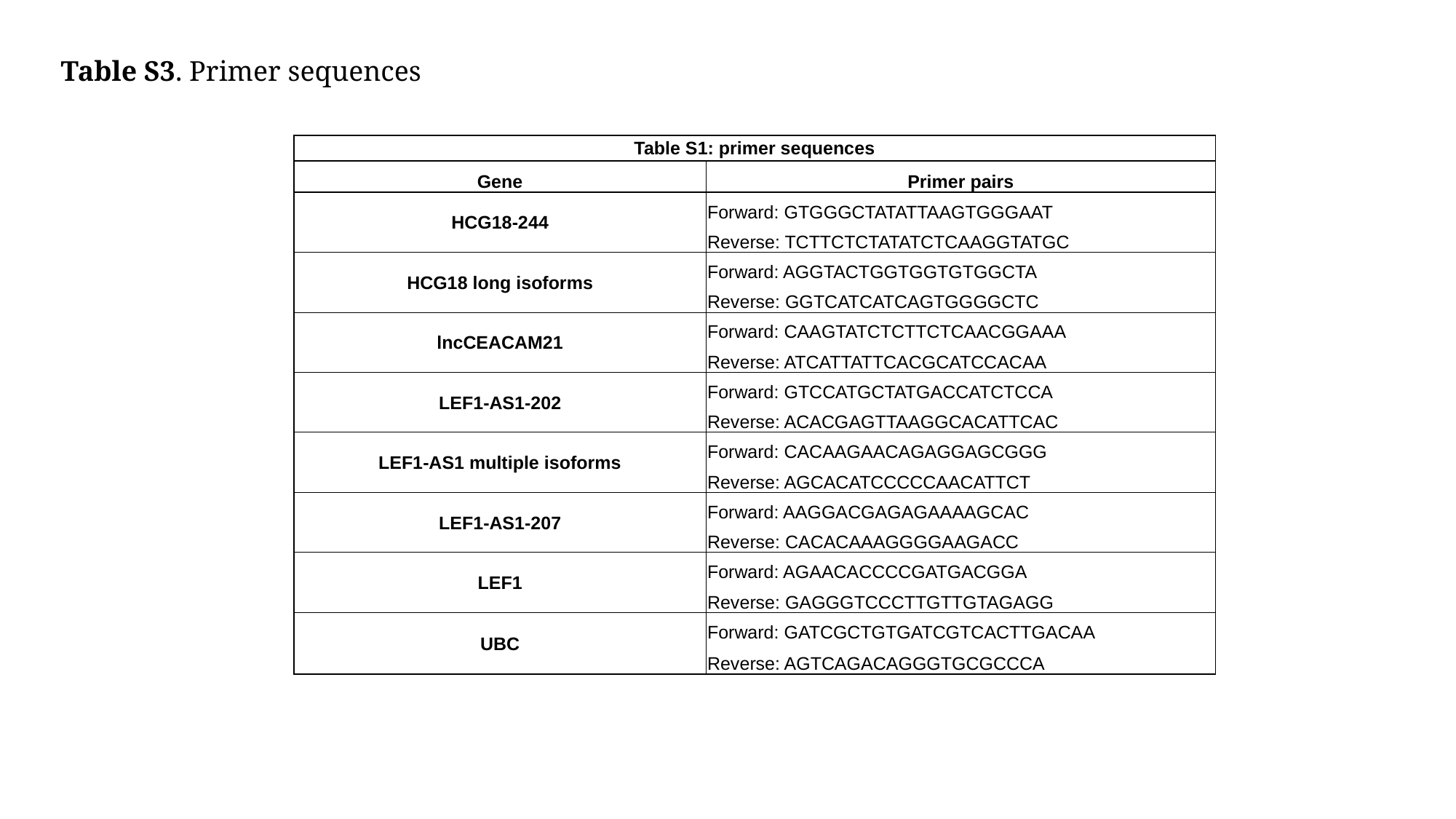

Table S3. Primer sequences
| Table S1: primer sequences | |
| --- | --- |
| Gene | Primer pairs |
| HCG18-244 | Forward: GTGGGCTATATTAAGTGGGAAT |
| | Reverse: TCTTCTCTATATCTCAAGGTATGC |
| HCG18 long isoforms | Forward: AGGTACTGGTGGTGTGGCTA |
| | Reverse: GGTCATCATCAGTGGGGCTC |
| lncCEACAM21 | Forward: CAAGTATCTCTTCTCAACGGAAA |
| | Reverse: ATCATTATTCACGCATCCACAA |
| LEF1-AS1-202 | Forward: GTCCATGCTATGACCATCTCCA |
| | Reverse: ACACGAGTTAAGGCACATTCAC |
| LEF1-AS1 multiple isoforms | Forward: CACAAGAACAGAGGAGCGGG |
| | Reverse: AGCACATCCCCCAACATTCT |
| LEF1-AS1-207 | Forward: AAGGACGAGAGAAAAGCAC |
| | Reverse: CACACAAAGGGGAAGACC |
| LEF1 | Forward: AGAACACCCCGATGACGGA |
| | Reverse: GAGGGTCCCTTGTTGTAGAGG |
| UBC | Forward: GATCGCTGTGATCGTCACTTGACAA |
| | Reverse: AGTCAGACAGGGTGCGCCCA |

## Slide 11
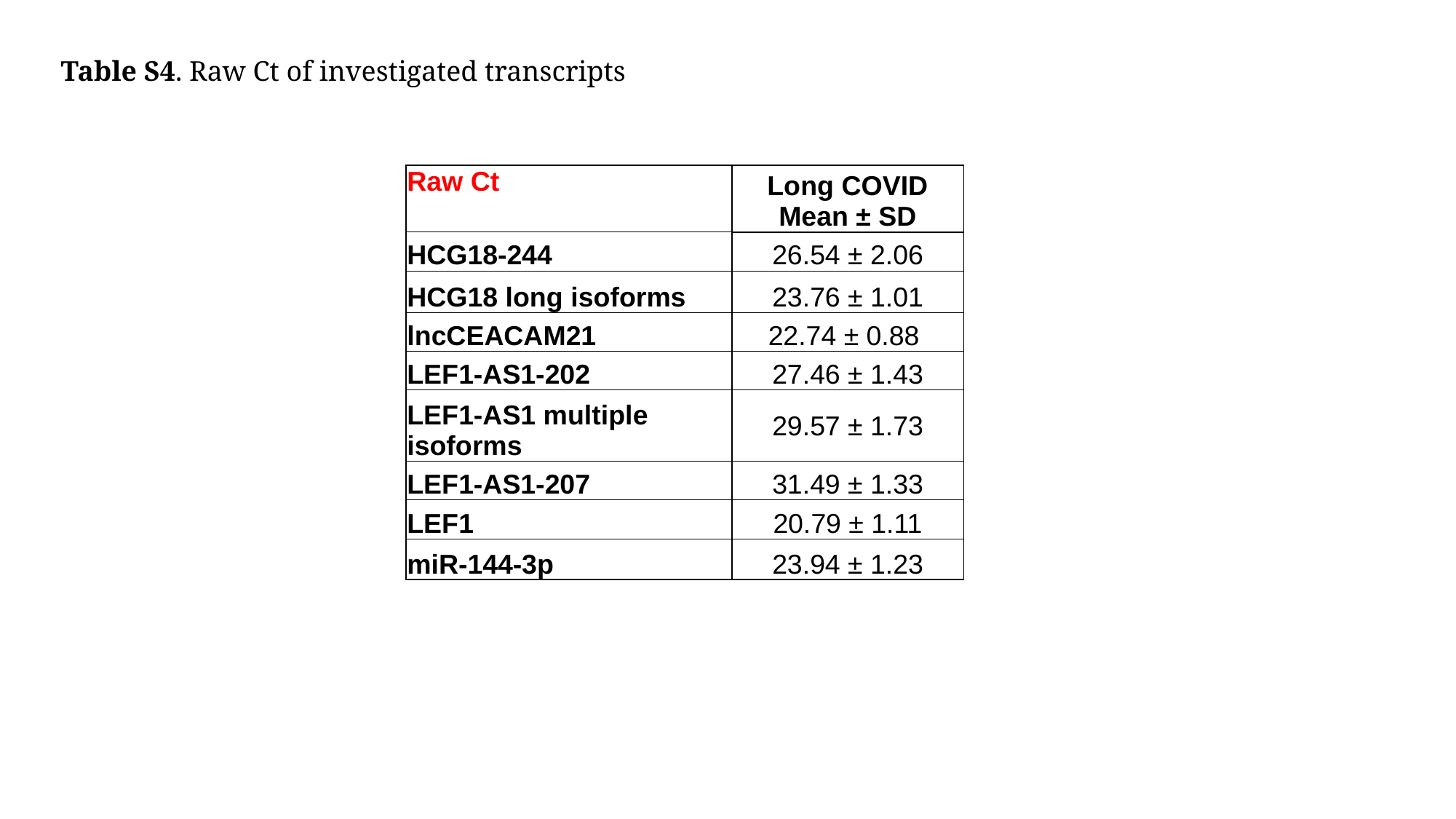

Table S4. Raw Ct of investigated transcripts
| Raw Ct | Long COVID Mean ± SD |
| --- | --- |
| HCG18-244 | 26.54 ± 2.06 |
| HCG18 long isoforms | 23.76 ± 1.01 |
| lncCEACAM21 | 22.74 ± 0.88 |
| LEF1-AS1-202 | 27.46 ± 1.43 |
| LEF1-AS1 multiple isoforms | 29.57 ± 1.73 |
| LEF1-AS1-207 | 31.49 ± 1.33 |
| LEF1 | 20.79 ± 1.11 |
| miR-144-3p | 23.94 ± 1.23 |

## Slide 12
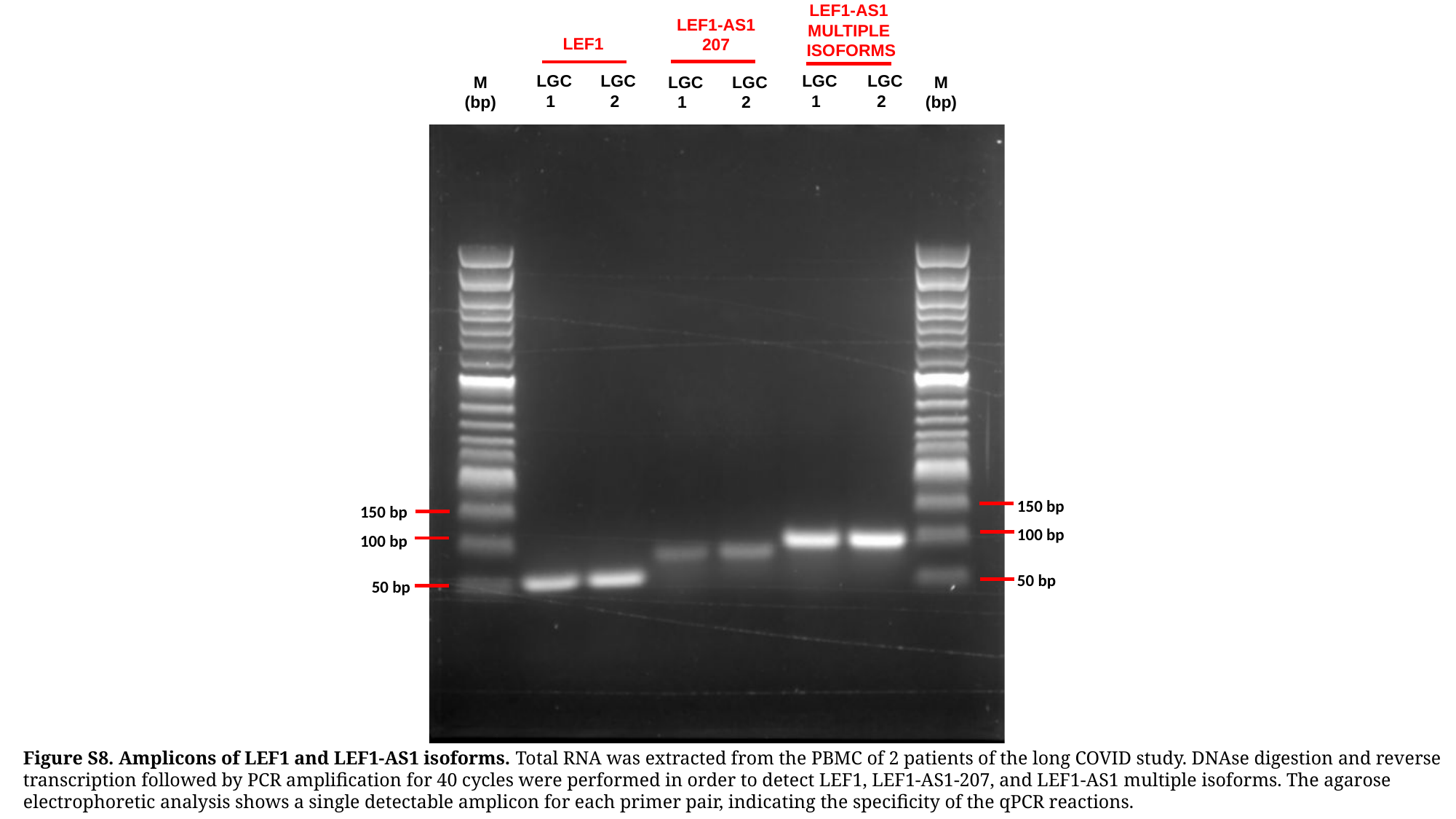

LEF1-AS1
MULTIPLE
 ISOFORMS
LEF1-AS1
207
LEF1
LGC
 1
LGC
 2
LGC
 1
LGC
 2
M
(bp)
M
(bp)
LGC
 1
LGC
 2
150 bp
150 bp
100 bp
100 bp
50 bp
 50 bp
Figure S8. Amplicons of LEF1 and LEF1-AS1 isoforms. Total RNA was extracted from the PBMC of 2 patients of the long COVID study. DNAse digestion and reverse transcription followed by PCR amplification for 40 cycles were performed in order to detect LEF1, LEF1-AS1-207, and LEF1-AS1 multiple isoforms. The agarose electrophoretic analysis shows a single detectable amplicon for each primer pair, indicating the specificity of the qPCR reactions.
